# Repeated Sulforaphane Treatment Reverses Depressive-like Behavior and Exerts Antioxidant Effects in the Olfactory Bulbectomy Model in Mice

**DOI:** 10.3390/ph17060762

**Published:** 2024-06-11

**Authors:** Patrycja Pańczyszyn-Trzewik, Katarzyna Stachowicz, Paulina Misztak, Gabriel Nowak, Magdalena Sowa-Kućma

**Affiliations:** 1 Department of Human Physiology, Institute of Medical Sciences, Medical College of Rzeszow University, Kopisto 2a, 35-959 Rzeszow, Poland; ppanczyszyn@ur.edu.pl; 2 Department of Neurobiology, Maj Institute of Pharmacology, Polish Academy of Sciences, Smetna 12, 31-343 Krakow, Poland; 3 Department of Medicine and Surgery, University of Milano-Bicocca, 20-900 Monza, Italy; 4 Department of Pharmacobiology, Jagiellonian University Medical College, Medyczna 9, 30-688 Krakow, Poland; 5 Centre for Innovative Research in Medical and Natural Sciences, Medical College of Rzeszow University, Warzywna 1A, 35-310 Rzeszow, Poland

**Keywords:** sulforaphane, Nrf2 activator, animal model of depression, olfactory bulbectomy, open field test, splash test, malondialdehyde, oxidative stress

## Abstract

Growing evidence suggests that activators of nuclear factor erythroid-derived 2-like 2 (Nrf2), such as sulforaphane, may represent promising novel pharmacological targets for conditions related to oxidative stress, including depressive disorder. Therefore, we conducted a study to explore the behavioral and biochemical effects of repeated (14 days) sulforaphane (SFN) treatment in the olfactory bulbectomy (OB) animal model of depression. An open field test (OFT), splash test (ST), and spontaneous locomotor activity test (LA) were used to assess changes in depressive-like behavior and the potential antidepressant-like activity of SFN. The OB model induced hyperactivity in mice during the OFT and LA as well as a temporary loss of self-care and motivation in the ST. The repeated administration of SFN (10 mg/kg) effectively reversed these behavioral changes in OB mice across all tests. Additionally, a biochemical analysis revealed that SFN (10 mg/kg) increased the total antioxidant capacity in the frontal cortex and serum of the OB model. Furthermore, SFN (10 mg/kg) significantly enhanced superoxide dismutase activity in the serum of OB mice. Overall, the present study is the first to demonstrate the antidepressant-like effects of repeated SFN (10 mg/kg) treatment in the OB model and indicates that these benefits may be linked to improved oxidative status.

## 1. Introduction

Depressive disorder (also known as depression) is the leading cause of the global health-related burden [1]. The World Health Organization (WHO) has indicated that approximately 280 million people worldwide suffer from depression [2]. Moreover, depression is a crucial risk factor for suicidal behavior, including suicidal ideation, suicide attempts, and completed suicide [3,4]. Unfortunately, the COVID-19 pandemic has also negatively affected mental health levels in society [5]. 

The cellular and molecular basis of depression pathophysiology is complex and remains largely unknown [6]. Another important aspect is the unsatisfactory effectiveness of antidepressants commonly used in the clinic and the high recurrence of depression [7]. Hence, it is essential to develop novel molecular targets for antidepressant therapies. 

Several human and experimental studies have confirmed the role of oxidative stress in the progression of depressive symptoms [8,9]. Our previous clinical studies have also revealed the role of inducing oxidative stress pathways in the pathogenesis of depression [10,11,12,13]. We also suggested that novel pharmacological treatments for depression should be based on antioxidant compounds [13]. In this context, nuclear factor erythroid-derived 2-like 2 (Nrf2), the primary endogenous regulator of the oxidative stress response, may play a key role in the neurobiology of depression and suicidality [14,15]. A postmortem study conducted by Martín-Hernández et al. (2018) showed decreased Nrf2 expression in the dorsolateral prefrontal cortex of major depressive disorder (MDD) patients [16]. In turn, our previous data indicated a significant reduction in Nrf2 protein levels in the frontal cortex (the nuclear fraction) of suicide victims [14]. Indeed, modulation of the intracellular Nrf2 pathway via specific activators may be a promising direction for the potentialization of depression therapy [15,17]. 

Among the Nrf2 activators, sulforaphane may have potential efficacy in the treatment of mental disorders [18]. However, the research on sulforaphane and depression is still in its early stages. We searched the PubMed database between 2013 and 2024 using the keywords *sulforaphane *depression. Only six citations in animal models of depression were identified, and they were mainly based on stress-induced or inflammatory paradigms. Notably, the specific molecular mechanism of sulforaphane’s antidepressant-like action remains to be elucidated and requires further investigation. In this context, bilateral olfactory bulbectomy (OB) is a well-validated animal model of agitated depression [19]. This model is based on the hypothesis that removing the olfactory bulbs (a part of the limbic system) affects their extensive efferent neuronal networks and disturbs the connection and function of the entire limbic system. OB induces behavioral, neurochemical, and neuromorphological changes in rodents, many of which mimic symptoms observed in depressed patients (good face validity) [20,21]. Importantly, OB causes oxidative stress, manifested as increased lipid peroxidation and decreased activity of Nrf2-related antioxidative enzymes [22,23,24]. In addition, Almeida et al. (2017) reported that OB leads to a long-lasting imbalance in the redox status of mice [25]. 

Thus, there is a lack of information regarding the effects of sulforaphane on behavioral and molecular regulation induced by OB. Therefore, this study examined the potential antidepressant-like activity of repeated administration of (R, S)-sulforaphane (SFN; 2.5, 5, and 10 mg/kg) in the OB mice model. The changes in depressive behavior and the potential antidepressant-like activity of SFN (ability to reverse behavioral abnormalities induced by OB) were evaluated using the open field test (OFT), splash test (ST), and spontaneous locomotor activity test (LA). In addition, in the biochemical analysis, we investigated the effects of OB and SFN (10 mg/kg) on the levels/activity of selected oxidative stress parameters (TBARS, thiobarbituric acid-reactive substances; TAC, total antioxidant capacity; and SOD, superoxide dismutase activity). Molecular studies were conducted in the frontal cortex (FCx), hippocampus (Hp), and serum of mice.

## 2. Results

### 2.1. Behavioral Studies 

#### 2.1.1. The Effects of the Olfactory Bulbectomy OB Procedure and Repeated SFN Administration on the Behavior of Mice in the Open Field Test

The OFT was performed at three time points (OFT1-3; see Section 4). The OFT1 results showed no differences in the basal locomotor activity of the SHAM and OB control groups (results not presented). The OFT2 results are included in the Supplementary Materials (Figure S1). 

**Figure 1:**
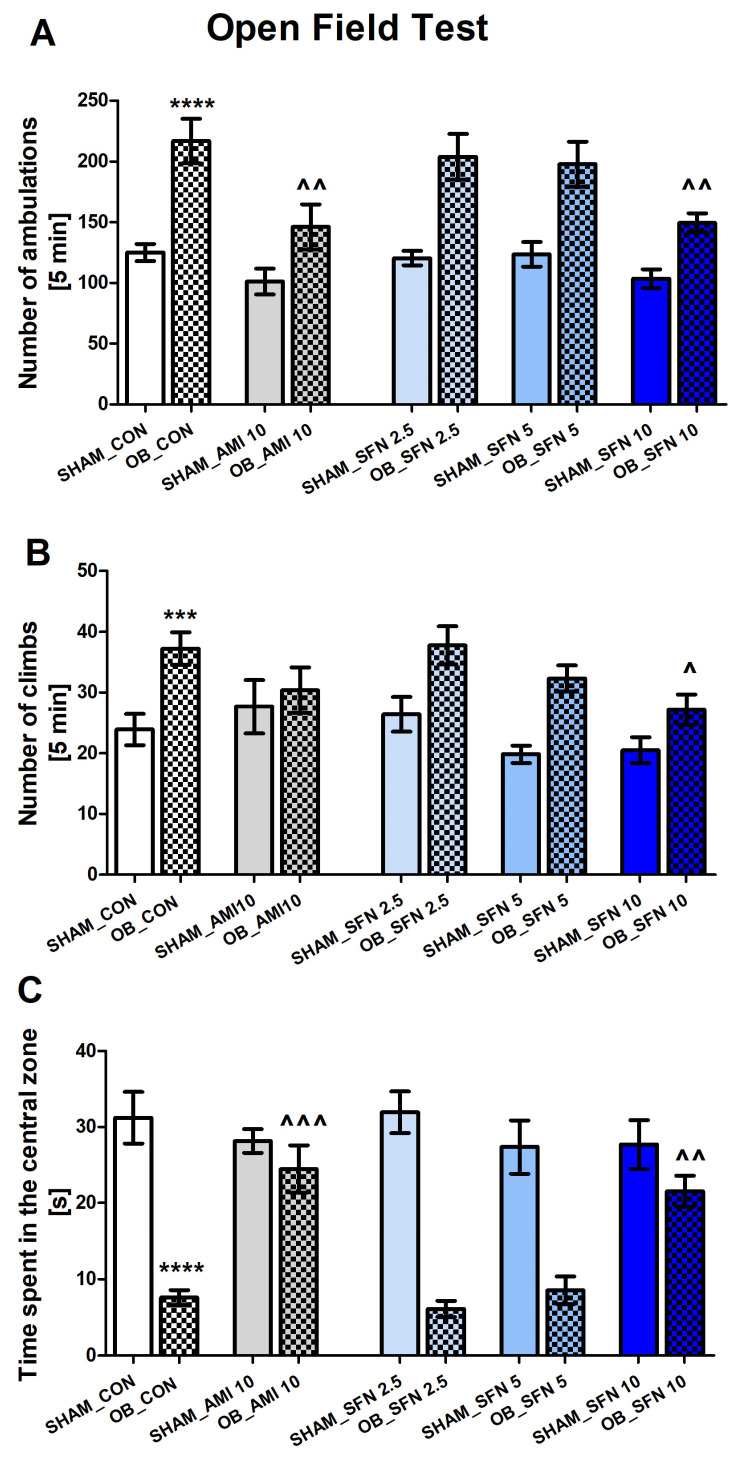
The effect of the OB procedure and repeated AMI (10 mg/kg) and SFN (2.5; 5 and 10 mg/kg) administration on the activity of mice in the open field test (OFT3; after a 14-day recovery period; day 30 of the experimental procedure). (**A**) number of ambulations, (**B**) number of climbs, and (**C**) time spent in the central zone. Data were analyzed using a two-way ANOVA and the Tukey Multiple Comparison test. *** *p* < 0.001, **** *p* < 0.0001 vs. SHAM_CON, ^ *p* < 0.05, ^^ *p* < 0.01, ^^^ *p* < 0.001 vs. OB_CON. Values are expressed as the mean ± SEM (*N* = 8–10).

The OB procedure induced the hyperactivity of mice, expressed as an increase in the number of ambulations (Figure 1A, *p* < 0.0001) and climbs (Figure 1B, *p* = 0.006) in the OFT3. Moreover, as shown in Figure 1C, mice subjected to the OB procedure presented statistically significant decreased time spent in the central zone of the OFT (*p* < 0.0001). A 14-day SFN (10 mg/kg) administration significantly reduced both ambulation (Figure 1A, *p* = 0.0055) and climb episodes (Figure 1B, *p* < 0.05) in bulbectomized mice. As shown in Figure 1A, amitriptyline (AMI as a reference drug) decreased the number of ambulations (*p* = 0.0061). Interestingly, SFN (10 mg/kg) and AMI effectively increased the time spent in the OFT central zone (Figure 1C; SFN10: *p* = 0.0026; AMI: *p* = 0.0002) in OB mice. As shown in Figure 1A–C, lower doses of SFN (2.5 or 5 mg/kg) had no effect in the OB group in parameters measured in OFT. At the same time, all tested compounds had no effect in the SHAM group. 

The two-way ANOVA results are as follows: number of ambulations [Figure 1A: effect of OB: F(1,97) = 67.28, *p* < 0.0001; effect of drugs administration: F(4,97) = 5.456, *p* < 0.0005; and no significant interaction: F(4,97) = 1.302, *p* = 0.2746], number of climbs [Figure 1B: effect of OB: F(1,88) = 29.79, *p* < 0.0001; effects of drugs administration: F(4,88) = 3.432, *p* = 0.0118; and no significant interaction: F(4,88) = 1.240, *p* = 0.2998], and time spent in the OFT central zone [Figure 1C: effect of OB: F(1,92) = 90.33, *p* < 0.0001; effect of drugs administration: F(4,92) = 4.018, *p* = 0.0048; and significant interaction: F(4,92) = 7.310, *p* < 0.0001].

#### 2.1.2. The Effects of OB and Repeated SFN Administration on the Motivational Behavior of Mice in the Splash Test 

The ST was performed at two time points (ST1-2; see Section 4). The ST1 results are included in the Supplementary Materials (Figure S2). 

**Figure 2:**
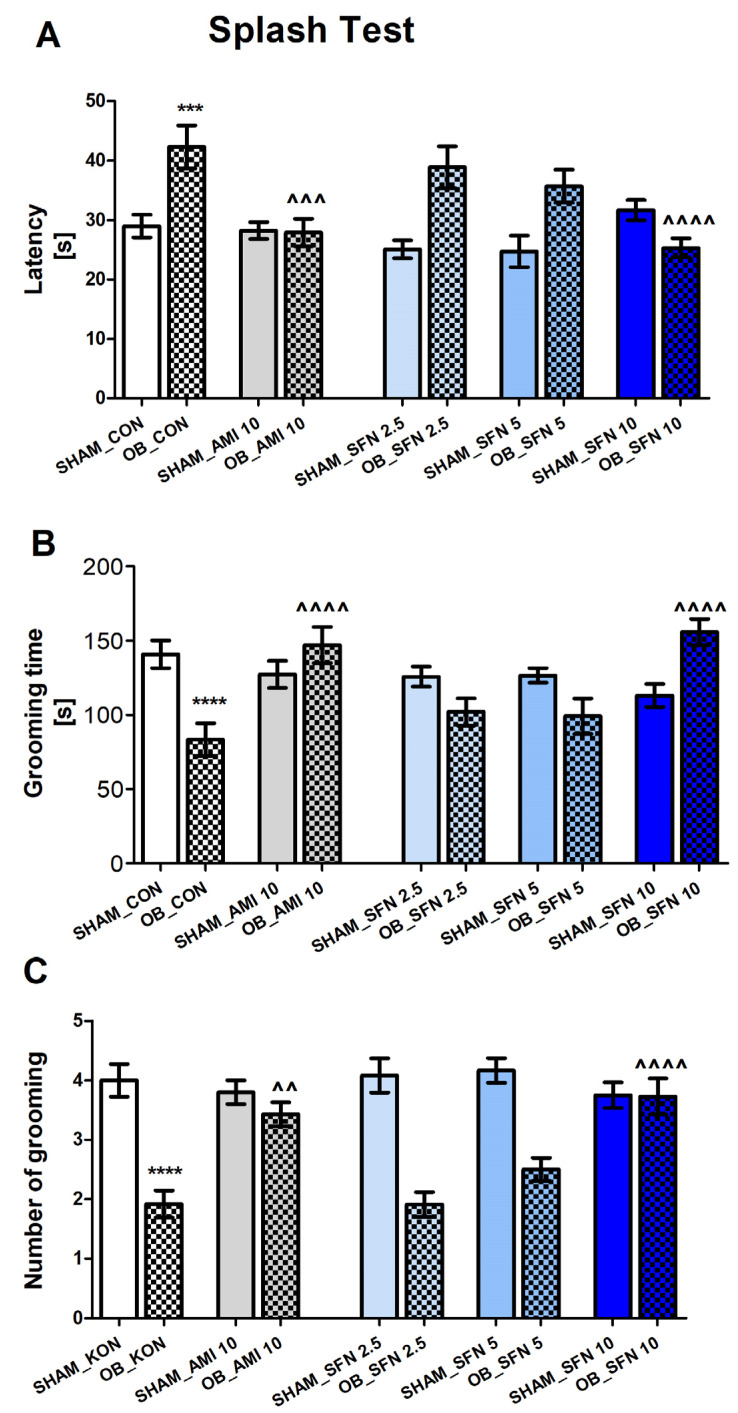
The effect of the OB procedure and AMI (10 mg/kg) and SFN (2.5; 5 and 10 mg/kg) administration on the index of self-care and motivational behavior of mice in the splash test (ST3; day 31 of the experimental procedure). (**A**) latency, (**B**) grooming time, and (**C**) number of grooming instances. Data were analyzed using a two-way ANOVA and the Tukey Multiple Comparison test. *** *p* < 0.001, **** *p* < 0.0001 vs. SHAM_CON, ^^ *p* < 0.01, ^^^ *p* < 0.001, ^^^^ *p* < 0.0001 vs. OB_CON. Values are expressed as the mean ± SEM (*N* = 8–10).

The OB procedure increased latency (Figure 2A, *p* = 0.0001) and decreased time and number of grooming instances (Figure 2B, *p* < 0.0001; Figure 2C, *p* < 0.0001, respectively). As shown in Figure 2A–C, repeated administration of SFN at the highest dose of 10 mg/kg (but not 2.5 or 5 mg/kg) and AMI (10 mg/kg) reversed these effects in OB mice, observed as significantly decreased latency (Figure 2A; SFN10 *p* < 0.0001; AMI *p* = 0.0007) increased time (Figure 2B; SFN10 *p* < 0.0001, AMI *p* < 0.0001) and number (Figure 2C; SFN10 *p* < 0001; AMI *p* = 0.0013) of grooming instances. All tested compounds had no effect in the SHAM group. 

The two-way ANOVA analysis results are as follows: latency [Figure 2A: effect of OB: F(1,95) = 17.69, *p* < 0.0001; effect of drugs administration: F(4,95) = 3.421, *p* = 0.0117; and significant interaction: F(4,95) = 7.852, *p* < 0.001], time of grooming [Figure 2B: no effect of OB: F(1,91) = 2.604, *p* = 0.1101; effect of drug administration: F(4,91) = 3.798, *p* = 0.0067); and significant interaction: F(4,91) = 10.07, *p* < 0.0001], and number of grooming instances [Figure 2C: effect of OB: F(1,101) = 66.93, *p* < 0.0001; effect of drugs administration: F(4,101) = 4.257, *p* = 0.0032; and significant interaction: F(4,101) = 8.436, *p* < 0.0001].

#### 2.1.3. The Effects of OB and Repeated SFN Administration in the Spontaneous Locomotor Activity Test

**Figure 3:**
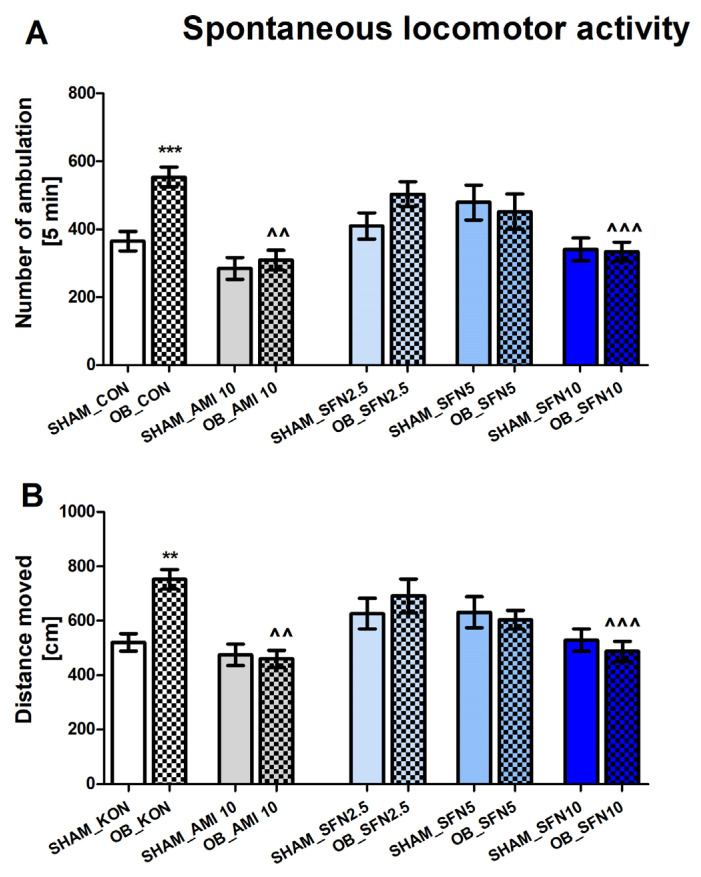
The effect of the OB procedure and repeated AMI (10 mg/kg) and SFN (2.5, 5, and 10 mg/kg) administration on spontaneous locomotor activity (day 32 of the experimental procedure). (**A**) number of ambulations, and (**B**) distance moved Data were analyzed using a two-way ANOVA and the Tukey Multiple Comparison test. ** *p* < 0.01, *** *p* < 0.001 vs. SHAM_CON, ^^ *p* < 0.01, ^^^ *p* < 0.001 vs. OB_CON. Values are expressed as the mean ± SEM (*N =* 8–10).

The effects of the OB procedure and repeated SFN administration were measured using the spontaneous locomotor activity test (LA) to exclude false-positive results in the OFT. In the OB group, a significant increase in the number of ambulations (Figure 3A, *p* = 0.009) and distance traveled (Figure 3B, *p* = 0.0011) was observed. As shown in Figure 3A,B, repeated SFN administration at a dose of 10 mg/kg (but not 2.5 or 5 mg/kg) and AMI (10 mg/kg) induced significantly reduced both ambulations (SFN10 *p* = 0.009, AMI *p* = 0.0018) and distance traveled (SFN10 *p* = 0.0009, AMI *p* = 0.0015) in OB mice. 

The two-way ANOVA analysis results are as follows: ambulations [Figure 3A: effect of OB: F(1,89) = 5.017, *p* = 0.0276; effect of drugs administration: F(4,89) = 8.143, *p* < 0.0001; and significant interaction: F(4,89) = 2.655, *p* = 0.0387] and distance traveled [Figure 3B: no effect of OB: F(1,83) = 2.081, *p* = 0.1529; effect of drugs administration: F(4,83) = 6.233, *p* = 0.0002; and significant interaction: F(4,83) = 2.882, *p* = 0.0274].

### 2.2. Biochemical Studies 

Only tissues from mice receiving the highest dose of SFN (10 mg/kg) were used for biochemical analyses, which was justified by the results of behavioral studies (see Section 2.1). Studies were conducted in the frontal cortex, hippocampus, and mouse serum to comprehensively evaluate the effect of Nrf2 activator administration at molecular levels.

#### 2.2.1. The Effects of the OB Procedure and Repeated SFN Administration on TBARS and TAC Levels in the FCx and Hp of Mice

**Figure 4:**
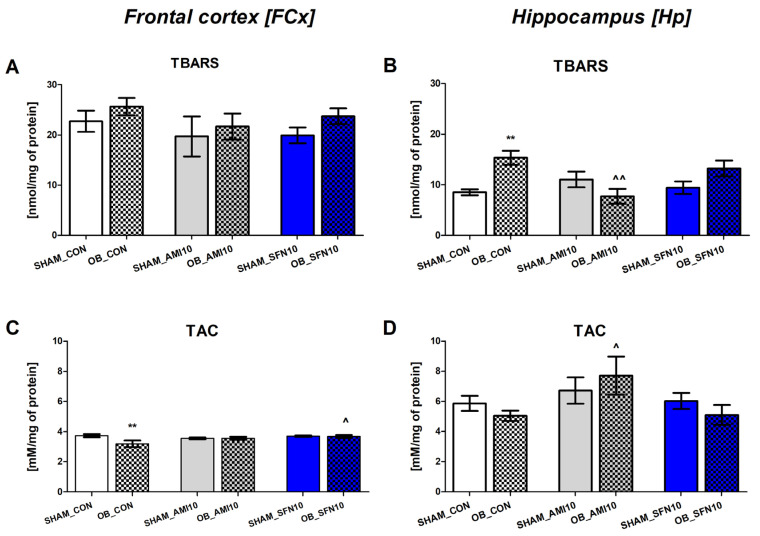
The effect of the OB procedure and repeated AMI (10 mg/kg) and SFN (10 mg/kg) administration on TBARS (**A**) and TAC (**C**) levels in the frontal cortex (FCx) and on TBRAS (**B**) and TAC (**D**) levels in the hippocampus (Hp). Data were analyzed using a two-way ANOVA and the Tukey Multiple Comparison test. ** *p* < 0.01 vs. SHAM_CON, ^ *p* < 0.05, ^^ *p* < 0.01 vs. OB_CON. Values are expressed as the mean ± SEM (*N* = 6–7).

The OB procedure significantly increased TBRAS levels in mice’s Hp (but not in the FCx) (Figure 4B, *p* = 0.0011). As shown in Figure 4B, the OB-induced increase in TBRAS levels was reversed by repeated AMI administration (10 mg/kg) (*p* = 0.0045). SFN showed only a statistically non-significant decrease in TBRAS levels in the Hp (*p* < 0.05). Therefore, a statistically significant reduction in TAC in the FCx (but not in the Hp) of OB mice was observed (Figure 4C, *p* = 0.0026). Repeated administration of SFN (but not AMI) reversed OB-induced TAC alterations (*p* = 0.0152). 

The two-way ANOVA analysis results for the FCx are as follows: [TBARS; Figure 4A: no OB effect: F(1,38) = 2.115, *p* = 0.1540; no effect of drugs: F(2,38) = 0.9775, *p* = 0.3855; and no significant interaction: F(2,28) = 0.07630, *p* = 0.9267]; [TAC: Figure 4C: no OB effect: F(1,35) = 3.581, *p* = 0.0667; no effect of drugs: F(2,35) = 1.948, *p* = 0.1578; and significant interaction: F(2,35) = 3.360, *p* = 0.046]. 

The two-way ANOVA analysis results for the Hp are as follows: [TBARS: Figure 4B: effect of OB: F(1,22) = 4.219, *p* = 0.04; no effect of drugs: F(1,22) = 1.703, *p* = 0.2052; and significant interaction: F(2,22) = 6.739, *p* = 0.0052); [TAC: Figure 4D: no OB effect: F(1,28) = 0.1546, *p* = 0.6972; effect of drugs: F(2,28) = 3.259, *p* = 0.03; and no significant interaction: F(2,28) = 0.9647, *p* = 0.3934]. 

#### 2.2.2. The Effects of the OB Procedure and Repeated SFN Administration on Serum Levels of TBARS and TAC, and SOD Activity

**Figure 5:**
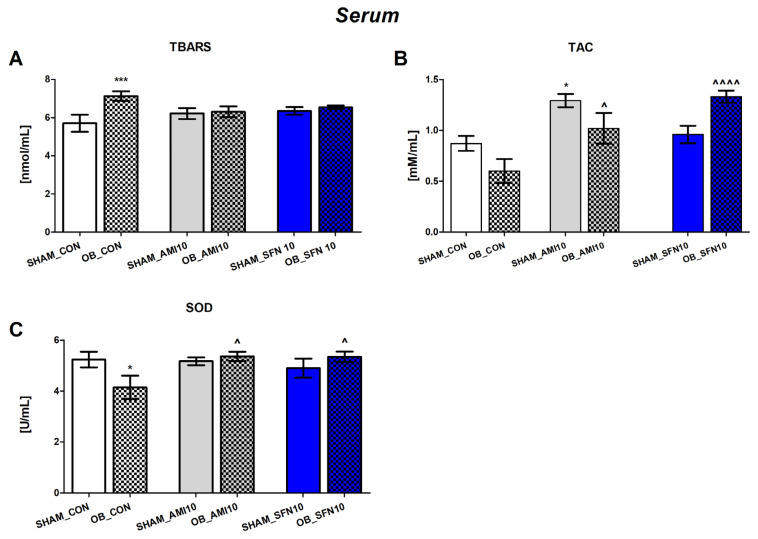
The effect of the OB procedure and repeated AMI (10 mg/kg) and SFN (10 mg/kg) administration on TBARS (**A**) and TAC (**B**) levels, and SOD activity (**C**) in serum. Data were analyzed using a two-way ANOVA and the Tukey Multiple Comparison test. * *p* < 0.05, *** *p* < 0.001 vs. SHAM_CON, ^ *p* < 0.05, ^^^^ *p* < 0.0001 vs. OB_CON Values are expressed as the mean ± SEM (*N* = 6–7).

The OB procedure induced a significant increase in TBARS levels (Figure 5A, *p* = 0.0009) and a decrease in SOD activity (Figure 5C, *p* = 0.0149). Repeated SFN and AMI administration caused significantly increased TAC levels (Figure 5B, SFN *p* < 0.0001; AMI *p* = 0.0130) and enhanced SOD activity (Figure 5C, SFN *p* = 0.0283; AMI *p* = 0.0257). As shown in Figure 5A, both SFN and AMI had no effects on serum TBARS levels in mice subjected to the OB procedure. 

The two-way ANOVA analysis results are as follows: TBARS [Figure 5A: effect of OB: F(1,36) = 6, *p* = 0.0166; no effect of drugs F(2,36) = 0, *p* = 0.7820; and significant interaction: F(2,36) = 3, *p* = 0.0387], TAC [Figure 5B: no OB effect: F(1,28) = 0.4928, *p* = 0.4885; effect of drugs: F(2,28) = 11.32, *p* = 0.0002; and significant interaction: F(2,28) = 6.797, *p* = 0.0039], and SOD [Figure 5C: no OB effect: F(1,34) = 0.3669, *p* = 0.5437; no effect of drugs: F(2,34) = 1.905, *p* = 0.1644; and significant interaction: F(2,34) = 3.632, *p* = 0.0372]. 

Furthermore, there was a strong positive correlation between serum TBARS levels and TBRAS levels in the Hp of all OB groups (control, AMI 10, and SFN 10; r = +0.8521–0.9415; *p* = 0.0312–0.0050; results presented in Table S1 in the Supplementary Materials). A Pearson correlation also revealed a positive correlation between serum TAC levels and changes in this parameter observed both in the FCx and the Hp of OB mice receiving SFN 10 (FCx: r = 0.9035; *p* = 0.0135; Hp: r = 0.9279; *p* = 0.0076, respectively).

## 3. Discussion 

Attention deficit hyperactivity disorder (ADHD) has been linked with psychiatric disorders [26,27]. A growing body of evidence suggests that the potential associations between ADHD and psychiatric disorders may vary based on gender. Solberg et al. (2018) showed that the prevalence of ADHD was significantly higher in women than in men for all psychiatric disorders [27]. These findings support the evaluation of motor deficits in studies using animal models of depression, such as the olfactory bulbectomy model (OB).

The current study demonstrated that OB induced hyperactivity in mice and decreased the time spent in the central zone of the open field test (OFT), mimicking symptoms of human depression. Our findings are consistent with previous studies and confirm that the olfactory bulb removal procedure was performed correctly [25,28,29]. The OFT is commonly used in animal research on the neurobiological basis of depression and anxiety, as well as for screening potential drug targets of these conditions [30,31]. Therefore, in subsequent behavioral studies, we investigated the potential antidepressant-like activity of (R, S)-sulforaphane (SFN), a potent exogenous activator of Nrf2. Our study also used amitriptyline 10 mg/kg (AMI) as a reference drug (positive control), which has a well-documented antidepressant effect in the OB model [32,33,34]. The OFT results showed that repeated daily administration of SFN at 10 mg/kg (but not at 2.5 or 5 mg/kg) reduced OB-induced hyperactivity, with an effect comparable to that of AMI. Furthermore, both SFN (10 mg/kg) and AMI significantly increased the time spent in the central zone of OFT. To ensure the accuracy of our findings, we took extra measures to eliminate false-positive results in the OFT. The mice’s spontaneous locomotor activity was measured, validating the OFT changes following the OB procedure and high-dose SFN (10 mg/kg) administration. These observations suggest the potential antidepressant effects of SFN in behavioral tests. Importantly, our results align with a study by Wu et al. (2016), demonstrating that SFN (10 mg/kg, i.p.; 14 days) significantly decreased immobility time in the forced swimming test (FST) and tail suspension test (TST) in acutely stressed mice. Similarly, SFN (10 mg/kg, i.p.; 14 days) increased immobility time in the FST and TST of mice subjected to a chronic mild stress (CMS) model [35]. Another study indicated the preventive effect of SFN (30 mg/kg, i.p.; single dose) on lipopolysaccharide (LPS)-induced depressive-like ve behavior in mice [36]. Additionally, SFN inhibited anxiety and depressive-like behaviors in mice with neuropathic pain at a dose of 10 mg/kg (i.p.; single dose) [37]. Moreover, a dietary intake of 0.1% glucoraphanin (a precursor of SFN) prevented depressive-like behavior after repeated social defeat stress [38]. These findings suggest that sulforaphane may be highly effective in reducing depressive-like ve behavior across various inducers. 

In the next step, we examined the effects of OB and repeated administration of SFN and AMI on motivational behavior in the splash test (ST), a test that enables the measurement of behavioral endpoints associated with the depressive anxiety phenotype in rodents [37]. Consistently, OB-induced behavioral effects, a significant increase in latency, and a decrease in time and number of grooming instances were observed in the ST. The results indicated a temporary loss of self-care and motivation in OB mice, typical of the depressive phenotype and anhedonia development [25,39,40]. Moreover, Almedia et al. (2017) showed for the first time that OB induced transient behavioral changes in the ST [25]. In turn, both SFN (10 mg/kg) and AMI reversed these effects in OB mice, significantly decreasing latency and increasing grooming time and number of instances. Previous studies have shown that antidepressant drugs or novel compounds with antidepressant and anxiolytic properties can reverse the effects induced by the OB procedure on motivational behavior in the ST [25]. For example, the selective serotonin reuptake inhibitor (SSRI) fluoxetine (10 mg/kg; p.o.; 14 days) reversed OB-induced changes in the ST, while chrysin (a flavonoid, 20 mg/kg, p.o.; 14 days) prevented a decrease in grooming time in OB mice [23,41,42,43]. These results correspond positively with the effects observed in the present study for SFN at a dose of 10 mg/kg and AMI, suggesting that SFN may regulate motivational behavior similarly to antidepressants.

In summary of behavioral studies, we have demonstrated for the first time that repeated administration of SFN for 14 consecutive days (10 mg/kg) promotes antidepressant-like effects in mice subjected to olfactory bulbectomy, with effects comparable to those of amitriptyline. The potential anxiolytic effects of SFN (10 mg/kg) observed in the OFT and ST require further investigation, such as confirmation in the elevated plus maze (EPM) test. 

The antioxidant mechanism of SFN is considered a crucial intracellular regulation that may play a significant role in the potential antidepressant activity of this compound. A growing body of evidence indicates that SFN could potentially reverse a depressive-like ve phenotype by primarily acting through the Nrf2 pathway, providing neuroprotective effects against oxidative damage [17]. To investigate the possible molecular mechanisms underlying the antidepressant-like action of SFN (10 mg/kg) in OB mice, we evaluated its effects on selected oxidative stress parameters levels/activity. Biochemical analyses were conducted on mouse brain tissue (frontal cortex—FCx and hippocampus—Hp, two key brain structures in the development of depressive-like behavior and sensitivity to oxidative stress) and serum. Due to the documented role of Nrf2 in regulating lipid peroxidation and stimulating global antioxidant activity, this study assessed the levels of peroxidation products (TBARS) and total antioxidant activity (TAC) [44]. The activity of superoxide dismutase (SOD) was additionally evaluated in the serum.

A significant increase in TBARS levels was observed in the Hp (but not the FCx) of OB mice. Jindal et al. (2015) similarly demonstrated enhanced lipid peroxidation (expressed as increased TBARS levels) in the Hp caused by olfactory bulbectomy [45]. OB mice contained significantly higher levels of TBARS in mitochondrial fractions from the Hp [46] Levels of 4-hydroxy-2-nonenal (4-HNE; a secondary product of lipid peroxidation) increased significantly in the Hp of bulbectomized mice [47]. These data indicate that OB is linked to the development of oxidative stress. Only AMI, not SFN, showed effects in reducing hippocampal TBARS levels in OB mice. SFN exhibited a non-significant decreasing trend. The observed findings of AMI are consistent with Leduc et al. (2002)’s study that indicates its effect on reducing TBRAS levels in inflammatory animal models [48]. In turn, there are currently no studies demonstrating the effects of SFN on TBARS levels in animal models of depression. The overall evidence suggests that sulforaphane may prevent lipid peroxidation by enhancing mitochondrial function or regulating Nrf2-related targets, such as glutathione peroxidase [49,50,51,52,53,54]. Indeed, our results cannot exclude the effect of SFN on lipid peroxidation. However, further molecular studies are required to determine the exact target of this action. Notably, lipid peroxidation is a complex biological process. In methodological terms, measuring TBARS and MDA levels/concentrations, commonly defined as potential indicators of oxidative damage to lipids, should be supplemented by assessing other non-enzymatic parameters involved in this process (such as F2-isoprostanes analysis). 

Furthermore, we evaluated the effects of OB and SFN on TAC to understand the potential antioxidant properties of SFN. Our analysis revealed that TAC levels in the FCx (but not in the Hp) were significantly lower in OB mice. Interestingly, SFN effectively increased TAC in both the FCx and serum. TAC is one of the most commonly measured parameters of redox signaling and provides an opportunity to evaluate antioxidant components [55]. Several studies reported lower TAC levels in acute depressive episodes of depressed patients [56]. Based on the obtained results, we can only speculate that SFN may act similarly to antidepressants by increasing total antioxidant capacity [57]. However, discussing TAC changes in conjunction with other specific antioxidant parameters is important. The increase in serum TAC levels and enhancement of superoxide dismutase (SOD) activity observed in the current study after repeated administration of SFN may be associated with metabolic mobilization and the upregulation of the Nrf2-dependent antioxidant pathway [58]. Protection against ROS-induced damage based on Nrf2 activation and upregulated SOD expression is well documented [59,60,61]. Our results also showed a different profile of changes in selected oxidative stress parameters depending on the brain region induced by the OB procedure and drug administration. The impact of Nrf2 regulation and SFN effects on the brain appear to be region-specific. Therefore, further studies are necessary to examine the role of the neurotrophin hypothesis in the hippocampus or the regulation of the hypothalamic–pituitary–adrenal axis in the frontal cortex on the Nrf2-SFN antioxidant response. From a methodological perspective, our study showed that potential SFN effects should be investigated both in the brain and serum. The obtained Pearson correlation results also justify this analysis scheme.

Despite the limitation mentioned above, our findings support the hypothesis that SFN has potential antidepressant and antioxidant properties. Moreover, impaired redox homeostasis may contribute to behavioral disturbances observed in OB mice. The antidepressant-like effects of SFN shown in behavioral studies seem to be associated with the effective inhibition of oxidative stress responses. Additionally, it can be asserted that SFN could be beneficial in preventing OB-induced oxidative stress. The changes observed in our biochemical studies require further detailed analysis, including determining the precise impact of SFN on molecular regulation in the Nrf2-dependent pathway, particularly in relation to its antioxidant, anti-inflammatory, and antidepressant effects.

## 4. Materials and Methods

### 4.1. Laboratory Animals and Housing

The experiments were carried out on male C57BL/J6 mice (~8 weeks old, 20–25 g) obtained from Charles River Laboratories (Erkrath, Germany). Upon arrival, the mice were habituated to the laboratory conditions for one week (acclimation period). During this time, the animals were periodically weighed and accustomed to contact with the experimenter to reduce any stress over planned experimental tasks. Throughout the whole experiment, the mice were kept under controlled laboratory conditions (12 h light/dark cycle; temperature: 22 ± 20 °C; humidity: 55 ± 5%) with food and water freely available. Each experimental group consisted of eight to ten animals. All procedures to which the animals were subjected (including behavioral tests) were carried out between 9:00 a.m. and 2 p.m.

The 3Rs (Replacement, Reduction, and Refinement) principle was considered when planning animal research. All procedures were conducted according to Polish law and approved by the 2nd Local Ethical Committee of the Maj Institute of Pharmacology of the Polish Academy of Sciences, Krakow (consent number 231/2017).

### 4.2. Olfactory Bulbectomy Procedure

After one week of acclimation, the animals were randomly divided into two groups: SHAM and OB-operated. In the planning stage, the total number of animals in the experimental groups was determined by analyzing the power of the ANOVA test (comparing more than two experimental groups) using G Power software version 3.1.9.7. In addition, years of research experience in planning experiments using the olfactory bulbectomy model have been considered. Despite extensive experience using the OB model, it is impossible to predict the mortality of animals during the experiment with high probability. It is well known that animals from the OB group are more likely to die earlier (even during surgery or as a result of complications after it). Moreover, after euthanasia, when collecting tissue for further tests, the correctness of the suction of the olfactory bulbs is verified. For these reasons, slightly larger groups of OB animals were planned in this experiment. In this study, all animals survived until its completion, and we found that the removal of the olfactory bulbs was 100% correct. This is why the final number of mice included in the behavioral studies differed between the SHAM and OB (8 vs. 10/group).

A bilateral olfactory bulbectomy procedure was performed according to the method described earlier [33]. Briefly, the animals were anesthetized with an aqueous solution of xylazine (6 mg/kg; i.p.; Biowet, Poland; 9 January 2013 SPC) and ketamine (100 mg/kg; i.p.; Biowet, Poland; 16 May 2016 SPC). Next, an incision was made in the skin overlying the skull. After exposure of the skull, 0.8–1.2 mm diameter holes were drilled according to the specified coordinates based on the stereotactic atlas of the mouse brain (4 mm forwards from Bregma and 1mm from the centerline) [62]. The olfactory bulbs were removed by suction, and the holes were filled with a hemostatic sponge (Spongostan Standard, Jonhson & Jonhson, New Brunswick, NJ, USA) to stop the bleeding. The surgical field was washed with an aqueous antibiotic solution (penicillin/streptomycin), and the skin was closed with an anti-inflammatory and anti-hemorrhagic surgical tissue adhesive (SurgiBond Tissue Adhesive, St. Vith, Belgium). Meloxicam (0.05 mg/kg, s.c., as an analgesic drug; Metacam, Boehringer, Ingelheim, Germany; 601307-09) was administered for the next 2–3 days after the surgery and regularly (for 4–5 days) the postoperative field was washed with Betadine (Egis, Warsaw, Poland; R/3619) solution to eliminate any infection. SHAM-operated animals (controls) were treated similarly, except the olfactory bulbs were left untouched. All animals were given 14 days to recover following surgery. During this period, they were handled daily by the experimenter to eliminate any aggressiveness that could otherwise arise. Following surgery, the animals’ condition was closely monitored.

### 4.3. Experimental Procedures

**Figure 6:**
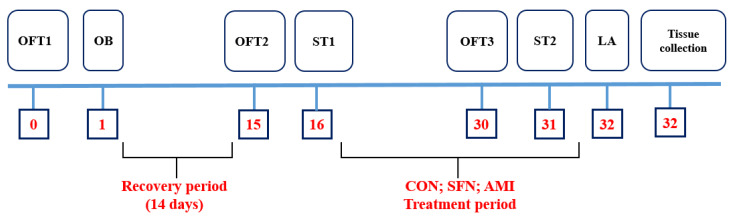
The schedule of the experimental procedure, including behavioral tests and the drug administration. After completion of behavioral testing, mice were euthanized by rapid decapitation. The brain tissue (frontal cortex and hippocampus) and serum were immediately collected for planned biochemical analyses. Abbreviations: OFT—open field test; OB—olfactory bulbectomy; ST—splash test; CON—control 1%DSMO; SFN—(R, S-sulforaphane) 2.5, 5 or 10 mg/kg, AMI—amitriptyline (10 mg/kg); LA—spontaneous locomotor activity test.

After a 2-week post-surgical period, the mice (both SHAM and OB) were divided into 10 experimental groups (see Table 1), and the administration of drugs was started. (R, S)-Sulforaphane (SFN: 2.5; 5 and 10 mg/kg; an Nrf2 activator; Abcam, Cambridge, UK; cat # ab141969) and amitriptyline (AMI: 10 mg/kg; reference drug; Sigma-Aldrich, Burlington, MA, USA; cat# A8404) were repeatedly administered once daily intraperitoneal (i.p.) for 14 consecutive days. The applied doses of the tested substances were chosen based on previously published studies [33,35,63]. SFN was dissolved in an aqueous solution of 1% dimethyl sulfoxide (DMSO; Sigma-Aldrich, Burlington, MA, USA; CAS # 67-68-5). The control groups received a 1% aqueous DMSO solution. After the 14th dose, the open field test was performed. Drug administration was continued for the next two days. Then, the splash and locomotor activity tests were conducted (Figure 6).

### 4.4. Open Field Test

The OFT was performed according to the procedure described by [42] with minor modifications. The “open field” (60 cm × 60 cm × 40 cm) apparatus consisted of a square arena in which the floor was divided into 12 equal squares. A 40 cm high wooden sheet surrounded the arena. The experiment was performed in a dark room, and the apparatus was illuminated by a 40 W bulb positioned 90–100 cm above the center of the arena. Each mouse was placed individually into the center of the “open field” apparatus, and the number of crossings (ambulation defined as the number of squares crossed by the animal using all paws) and number of climbs (number of times the mice stood on its hind legs or engaged in vertical exploratory activity) were measured for 5 min. Moreover, the time in the central zone to verify potential anxiety-like behaviors of the mice was recorded [31]. The apparatus was cleaned with a solution of 10% ethanol between trials to remove animal odors. The OFT was performed at three time points. The first test (OFT1) was performed to exclude possible individual differences among the animals. The second (OFT2) was conducted after 14 days of OB but before the start of drug administration to verify depressive-like behavior induced by the procedure. The last (OFT3) test was performed after the end of the drug administration.

### 4.5. Splash Test

The ST was adapted from [25]. In short, a high-viscosity 10% sucrose solution was sprayed on the dorsal coat of the mice to stimulate self-grooming behavior, defined as cleaning the fur by licking or scratching. After applying the 10% sucrose solution, the latency to grooming, number of instances, and total grooming time were measured for a total period of 5 min. These parameters were an index of self-care and motivational behavior of mice [42]. The ST was performed at two times points. ST1 was conducted after 14 days of OB but before the start of drug administration to verify depressive-like behavior induced by the procedure. ST2 was performed after the end of the drug administration.

### 4.6. Spontaneous Locomotor Activity Test

LA was assessed using an Opto-M3 Auto-Track (Columbus Instruments, Columbus, OH, USA). The Opto-M3 system consists of 20 cages with horizontal axes (X and Y) sensors connected to a computer. The sensor system allows the assessment of ambulation (activity counts) and the distance the animal moves. Locomotor activity was evaluated for 5 min, corresponding with the time interval analyzed in the OFT.

### 4.7. Tissue Collection

24 h after the last dose of drug administration (after 4 p.m.), at the end of the experiment, the mice were euthanized by rapid decapitation. The brain structures (the frontal cortex, FCx; and hippocampus, Hp) were immediately collected for planned biochemical analyses. The tissues were frozen on dry ice and next stored at −80 °C. At the same time, trunk blood was collected into tubes without anticoagulant. Next, the blood samples were allowed to clot for 15–20 min and centrifuged for 30 min at 1800× *g* at 4 °C. Afterward, the final supernatant (serum) was quickly pipetted into fresh tubes stored at −80 °C until analysis.

All mice were euthanized at the end of the experiment, and it was visually confirmed that two-thirds of the OB area had been lesioned. SHAM operations followed the same surgical procedure without the removal of the OB.

### 4.8. Oxidative Stress Assays

In the biochemical research (taking into account the need for a comprehensive assessment of the antioxidant profile and limited amount of mouse tissue, specifically brain structures—FCx, HP, and serum), we selected proportionally smaller groups, i.e., *n* = 6 and *n* = 7, for the SHAM and OB groups, respectively.

Oxidative parameters were measured using commercially available kits according to the manufacturer’s protocols. The levels of lipid peroxidation and total antioxidant capacity (TAC) were determined in the whole brain tissue (FCx, Hp) lysates and serum using a thiobarbituric acid-reactive substances (TBARS) Assay kit and Antioxidant Assay Kit (Cayman Chemical, Ann Arbor, MI, USA; cat #10009055; 700870), respectively. The malondialdehyde (MDA)-thiobarbituric acid (TBA) adduct was measured spectrophotometrically at 530 nm to determine lipid peroxidation. In turn, the Antioxidant Assay Kit was based on the ability of antioxidants in the sample to inhibit the oxidation of ABTS^®^ (2,2′-Azino-di-[3-ethylbenzthiazoline sulphonate]) to ABTS^®^•+ by metmyoglobin. The final product was monitored by reading the absorbance at 750 nm. Moreover, in serum, the superoxide dismutase activity (SOD) was estimated using a Superoxide Dismutase Assay Kit (Cayman Chemical, Ann Arbor, MI, USA; cat #706002). The SOD Assay Kit uses a tetrazolium salt for the detection of superoxide radicals generated by xanthine oxidase and hypoxanthine. One unit of SOD is defined as the amount of enzymes needed to exhibit 50% of the dismutation of the superoxide anions (U/mL). The absorbance was measured at 450 nm.

Values obtained for TBARS and TAC in whole tissue lysates were normalized to total protein concentration, which was determined using a PierceTM BCA Protein Assay Kit (ThermoFisher Scientific, Waltham, MA, USA).

### 4.9. Statistical Analysis

All data were expressed as the mean ± SEM. The results were analyzed using a two-way ANOVA followed by the Tukey Multiple Comparison test in GraphPad Prism 10 software. The Pearson correlation coefficient assessed correlations between quantitative variables. *p* < 0.05 was considered statistically significant.

## 5. Conclusions

Our data confirmed that OB causes depressive-like behavior associated with enhanced oxidative stress. This study demonstrated for the first time that repeated administration of SFN at 10 mg/kg, similar to AMI, reversed OB-induced changes in both behavioral and biochemical parameters. Our findings also suggest that the observed antidepressant effect of SFN may be associated with beneficial oxidative status outcomes. Importantly, our data showed the regulation of Nrf2 expression via a specific activator underlying agitated behavior. Indeed, agitated depression characterizes intensified suicidal trends. Our postmortem study also reported that the pathogenesis of suicidality is linked with Nrf2-related pathways [14]. However, additional molecular studies are necessary to elucidate the potential role of SFN and Nrf2 activation in suicide-related disorders such as depression. Further molecular studies that can promote the therapeutic effectiveness of SFN in the pharmacological treatment of mental disorders should assess the role of this Nrf2 activator in regulating CREB-BDNF-Trkβ signaling pathways, inflammatory responses, the NMDA-AMPA receptor complex, and non-apoptotic cell death mechanisms.

## Figures and Tables

**Table 1:** Division of animals into experimental groups.

SHAM	OB
Control–1% DMSO	Control–1% DMSO
(R, S)-sulforaphane 2.5 mg/kg	(R, S)-sulforaphane 2.5 mg/kg
(R, S)-sulforaphane 5 mg/kg	(R, S)-sulforaphane 5 mg/kg
(R, S)-sulforaphane 10 mg/kg	(R, S)-sulforaphane 10 mg/kg
Amitriptyline 10 mg/kg	Amitriptyline 10 mg/kg

## Data Availability

All data related to this study are available from the corresponding author and can be provided upon request.

## Supplementary Materials

The following supporting information can be downloaded at https://www.mdpi.com/article/10.3390/ph17060762/s1, Figure S1: The effect of the OB procedure on the activity of mice in the open field test (OFT2; after a 14-day recovery period; day 14 of the experimental procedure); Figure S2: The effect of the OB procedure on the index of self-care and motivational behavior of mice in the splash test (ST1; after a 14-day recovery period; day 15 of the experimental procedure), and Table S1: Pearson correlation coefficients.
