# Supplementary material for: Repeated Sulforaphane Treatment Reverses Depressive-like Behavior and Exerts Antioxidant Effects in the Olfactory Bulbectomy Model in Mice
Source: Pharmaceuticals (Basel). 2024 Jun 11;17(6):762. doi: 10.3390/ph17060762 (PMC11206991; doi:10.3390/ph17060762)
Supplement: Supplementary file 1 [file pharmaceuticals-17-00762-s001.zip › pharmaceuticals-3002419-supplementary.pdf]

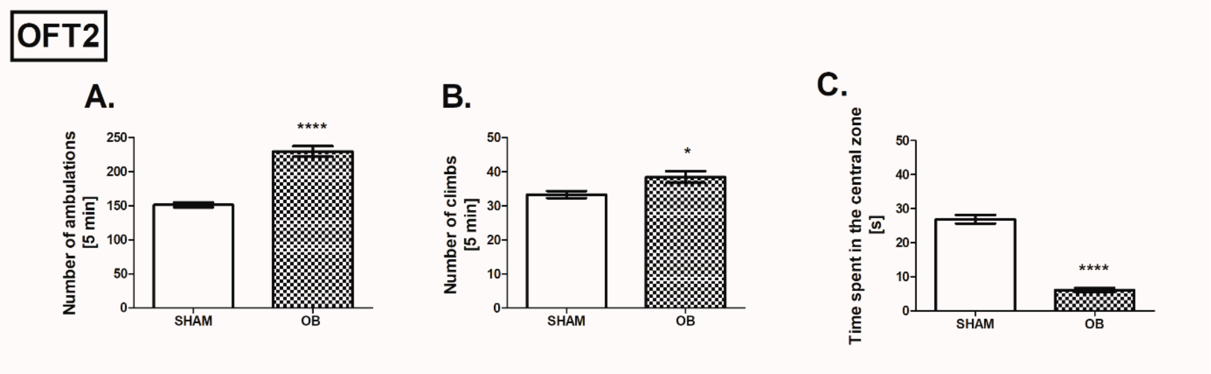


Figure S1: The effect of the OB procedure on the activity of mice in the open field test (OFT2; after a 14-day recovery period; day 14 of the experimental procedure). A: number of ambulations, B: number of climbs, C: time spent in the central zone. Data were analyzed by Student's t-test. *p<0.05, ****p<0.0001vs. SHAM_CON. Values are expressed as the mean ± SEM (N=8-10). Statistical details: number of ambulations [p<0.0001, t(110)=9.476]; number of climbs [p=0.0117, t(113)=2.563]; time spent in the central zone [p<0.0001, t(102)=14.98]

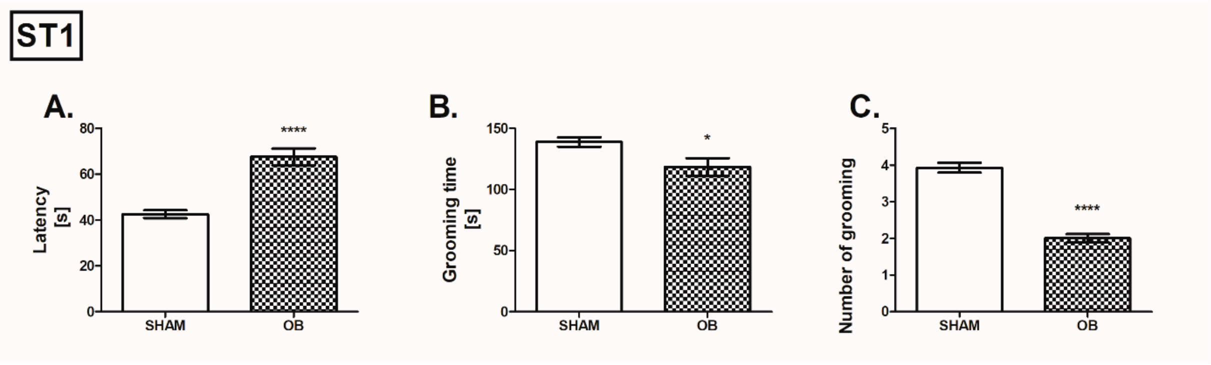


Figure S2: The effect of the OB procedure on the index of self-care and motivational behavior of mice in the splash test (ST1; after a 14-day recovery period; day 15 of the experimental procedure). A: latency, B: grooming time, C: number of grooming) Data were analyzed by Student's t-test. *p<0.05, ****p<0.0001vs. SHAM_CON. Values are expressed as the mean ± SEM (N=8-10). Statistical details: latency [p<0.0001, t(101)=6.043); grooming time [p=0.0110, t(89)=2.598]; number of grooming [p<0.0001, t(105)=10.90].

Table S1: Pearson correlation coefficients

| | group | r | p |
|---|---|---|---|
| TBARS serum vs. TBARS FCx | OB Control | 0.7255 | 0.0650 |
| | OB AMI 10 | 0.7478 | 0.0533 |
| | OB SFN 10 | 0.8281 | 0.0214* |
| TBARS serum vs. TBARS Hp | OB Control | 0.8521 | 0.0312* |
| | OB AMI 10 | 0.9204 | 0.0093** |
| | OB SFN 10 | 0.9415 | 0.0050** |
| TAC serum vs. TAC FCx | OB Control | 0.8730 | 0.0232* |
| | OB AMI 10 | 0.7998 | 0.0561 |
| | OB SFN 10 | 0.9035 | 0.0135* |
| TAC serum vs. TAC Hp | OB Control | 0.6175 | 0.1914 |
| | OB AMI 10 | 0.6883 | 0.1306 |
| | OB SFN 10 | 0.9279 | 0.0076** |

TBARS – thiobarbituric acid-reactive substances; TAC -; total antioxidant capacity; FCx- frontal cortex; Hp - hippocampus; OB – olfactory bulbectomy; SFN - (R, S)-sulforaphane at dose 10 mg/kg; AMI – amitriptyline at dose 10 mg/kg
